# CNN Sample-Size Effects Across Biomedical Datasets: A Reliability Pattern in Overfitting, Ranking, and Monotonicity

**DOI:** 10.3390/bioengineering13090971

**Published:** 2026-08-25

**Authors:** Giacinto Angelo Sgarro, Melle Mendikowski, Domenico Santoro, Luca Grilli

**Affiliations:** 1 Department of Economics, University of Foggia, 71121 Foggia, Italy; giacintoangelo.sgarro@unifg.it; 2 German Research Center for Artificial Intelligence (DFKI), 23562 Lübeck, Germany; melle.mendikowski@dfki.de; 3 Computer Science Department, University of Hamburg, 22527 Hamburg, Germany; 4 Department of Economics, Statistics and Business, Faculty of Technological and Innovation Sciences, Universitas Mercatorum, 00186 Rome, Italy; domenico.santoro@unimercatorum.it

**Keywords:** convolutional neural networks, deep learning, biomedical image classification, sample size, overfitting, architecture screening, rank stability, learning curves

## Abstract

Convolutional neural networks (CNNs) are widely used for biomedical image classification, yet it remains unclear under which conditions training on reduced subsets of available data can provide reliable guidance during model development, how much training data is required to achieve stable and comparable performance across CNN architectures, and whether increasing the training set size always leads to improved generalization or can sometimes result in degraded performance. We study this question across four biomedical datasets (breast mammography, pediatric chest X-ray, brain tumor MRI, and skin lesion dermoscopy) using the full grid of 39 CNN architectures (1–3 convolutional layers, 16/32/64 filters) from our companion architectural study, training each configuration from scratch on seven proportions of the training data (5%, 10%, 20%, 40%, 60%, 80%, and 100%) over 5 independent runs per configuration, with the test set held at a fixed size across all sample-size conditions to ensure a like-for-like comparison of generalization performance. The analysis investigates three complementary aspects of sample-size sensitivity: the stabilization of the training–test generalization gap as training-set size increases, the reliability of architecture rankings obtained from reduced training fractions as a proxy for the full-dataset ranking, and the monotonicity of test performance with respect to training-set size. Taken together, the results point to a rough four-band pattern of reliability across the sampled fractions—unstable below 20% of the training set, of uncertain overfitting status between 20% and 60%, comparatively stable between 60% and 80%, and potentially counterproductive beyond 80%—while showing that this pattern is itself dataset-dependent and offers no guarantee on architecture ranking, arguing against reduced-fraction screening as a reliable shortcut for CNN architecture selection in biomedical imaging. All code and datasets are publicly released for reproducibility.

## 1. Introduction

Convolutional Neural Networks (CNNs) are among the most widely used deep learning models for biomedical image classification, achieving remarkable performance across a broad range of imaging modalities [1,2,3,4,5,6]. Despite these advances, developing an effective CNN remains a computationally demanding process. Given the unique characteristics of biomedical datasets [7,8,9], practitioners are faced with two competing objectives: exploring a sufficiently broad range of training conditions to identify reliable performance patterns, while minimizing the number of simulations required to keep the associated computational cost manageable [10,11]. In practice, multiple architectures and hyperparameter combinations are typically evaluated before selecting the final model, requiring repeated training on large datasets to identify the best-performing configuration for practical use.

When designing CNN pipelines, practitioners routinely encounter practical questions that lack systematic empirical answers. One common practice is to train on a deliberately small subset of the available data to quickly verify that the implementation behaves as expected and that the model can successfully fit the training samples [12]. Another frequent strategy is to use reduced training fractions to obtain a preliminary ranking of candidate architectures, thereby avoiding the computational cost of training every configuration on the full dataset [13,14,15]. A third, more subtle question is whether using the entire training set is always beneficial, or whether increasing the amount of training data can, under some circumstances, lead to worse test performance [16,17,18,19].

These questions point to three complementary aspects of sample-size sensitivity, which need not behave consistently with one another or across datasets: whether the training–test generalization gap eventually stabilizes as the training-set fraction *p* increases, rather than continuing to widen [20,21]; whether the relative ranking of candidate architectures by test performance, obtained from a reduced fraction, is a reliable proxy for the ranking obtained on the full dataset [15,22,23]; and whether test accuracy is non-decreasing as the training set grows, or can instead decrease significantly beyond some point for specific architectures or datasets [16,17,18]. Existing studies typically report results for a single, fixed training-set size, often relying on data augmentation or transfer learning to compensate for limited data [24,25,26,27,28,29,30], and consequently provide little empirical guidance on any of these three aspects, or on whether they can be reduced to simple, dataset-independent rules of thumb [31].

We investigate these questions using the same family of CNN architectures, with increasing depth and filter width, introduced in our companion architectural study. Each architecture is trained from scratch on four biomedical imaging benchmarks: mammography (CBIS-DDSM) [32], brain MRI for tumor analysis [33], pediatric chest X-ray for pneumonia [34], and dermoscopic skin lesion imaging (ISIC 2017) [35]. For every dataset, a separate training subset is independently sampled at each of seven fractions of the original training set (5%, 10%, 20%, 40%, 60%, 80%, and 100%), with each architecture–sample-size combination evaluated over 5 independent runs, while keeping the test set fixed across all sample-size conditions to allow a like-for-like comparison of generalization performance.

All source code, dataset links, and trained-model configurations used in this study are publicly available at https://github.com/hyacintus/CNN-Architectural_Design-Biomedical-Datasets.git (accessed on 20 August 2026).

The remainder of this paper is organized as follows. Section 2 reviews the fundamental concepts of CNNs relevant to overfitting and generalization. Section 3 describes the four datasets. Section 4 details the experimental design, including the fixed hyperparameters, the CNN architectures considered, and the sample-size reduction procedure. Section 5 presents the sample-size sensitivity and overfitting analysis. Section 6 reports the results, Section 7 discusses their practical implications, and Section 8 concludes the paper.

## 2. Fundamentals of Convolutional Neural Networks

Convolutional Neural Networks (CNNs) are a class of deep learning models specifically designed for image analysis [36]. Unlike traditional machine learning approaches based on manually engineered features, CNNs automatically learn hierarchical representations directly from raw pixel data [37]. This capability makes them particularly effective in biomedical imaging, where relevant diagnostic information may depend on subtle morphological, structural, or textural patterns that are difficult to characterize manually [38].

The architecture of a CNN can be conceptually divided into a feature extraction component, composed of convolutional operations and nonlinear transformations, and a classification component that maps the extracted representations to the final prediction. Convolutional layers apply learnable filters to progressively extract increasingly abstract features from input images, while activation and pooling layers introduce nonlinearity and progressively reduce the spatial dimensionality of the learned representations [38,39].

**Figure 1:**
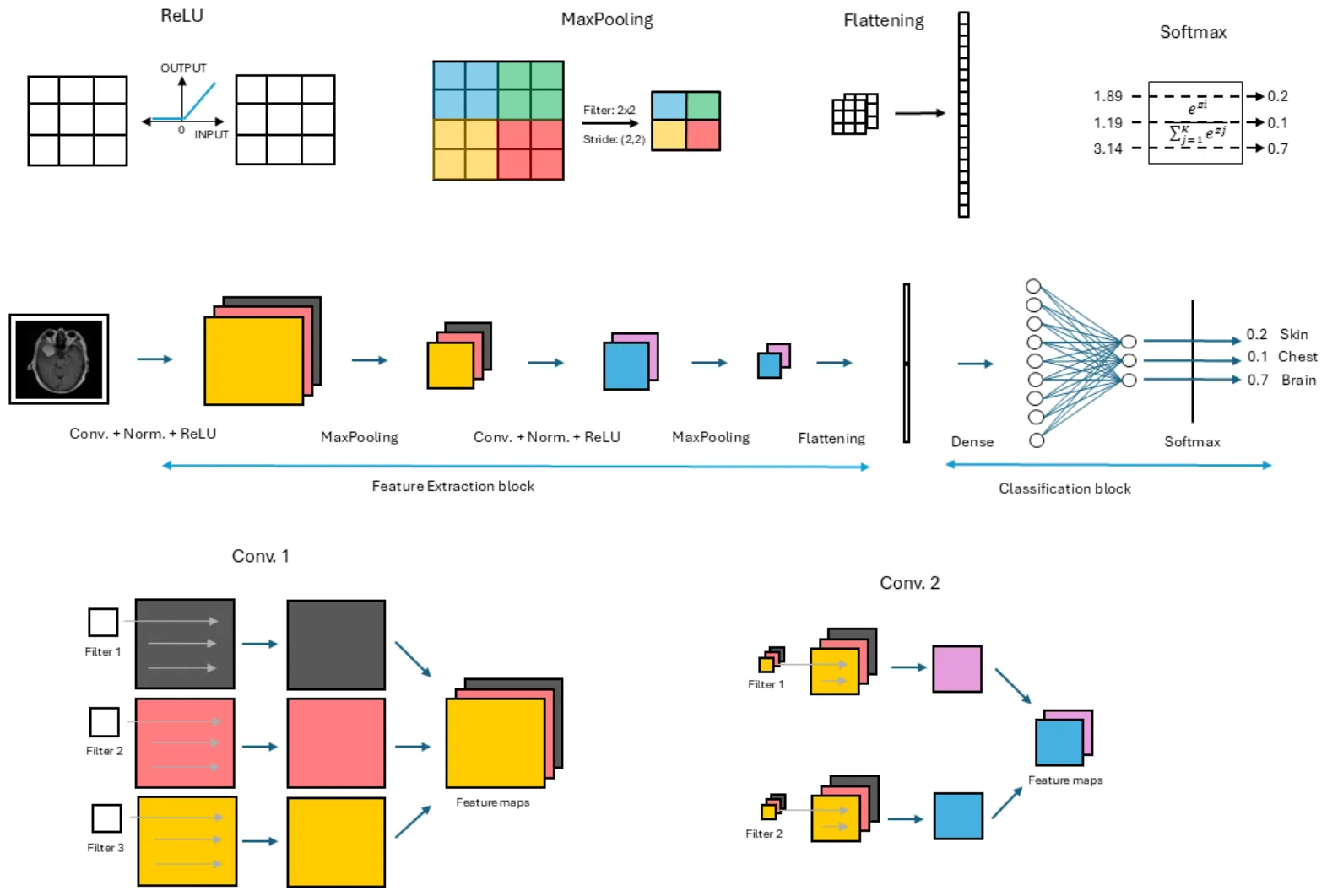
General schematic representation of a Convolutional Neural Network (CNN). Input images are processed through convolutional, activation, and pooling layers to extract hierarchical feature representations, which are subsequently used by fully connected layers for classification.

Figure 1 illustrates the general workflow of a Convolutional Neural Network, from feature extraction to the final classification. Images are processed through successive convolutional, activation, and pooling layers to extract hierarchical features that are subsequently used for classification.

The depth and width of a CNN determine its representational capacity through the number of trainable parameters [38]. Higher capacity does not necessarily imply better predictive performance, as model effectiveness depends on the interaction between architecture, dataset characteristics, and the amount of available training data [9,10].

CNN training consists of iteratively updating the model parameters to minimize the discrepancy between predictions and ground-truth labels. Parameter updates are computed by backpropagating the prediction error and optimizing the resulting gradients using algorithms such as stochastic gradient descent or Adam [39,40].

Model performance is evaluated using classification accuracy, one of the most widely adopted metrics for image classification tasks. Classification accuracy is defined as

(1) $$\mathrm{Acc}=\frac{TP+TN}{TP+TN+FP+FN},$$

where TP, TN, FP, and FN denote the numbers of true positives, true negatives, false positives, and false negatives, respectively. For datasets with three or more classes, accuracy is computed by aggregating the class-specific true positives and false negatives:

(2) $$\mathrm{Acc}=\frac{\sum_{c}TP_{c}}{\sum_{c}TP_{c}+\sum_{c}FN_{c}},$$

where *c* indexes the classes. In a multiclass confusion matrix, the class-specific true positives correspond to the diagonal elements, as illustrated in Table 1.

The sum of the diagonal elements therefore represents the total number of correctly classified samples, while the denominator corresponds to the total number of samples.

## 3. Applications of Convolutional Neural Networks to Four Biomedical Datasets

The proposed analysis is conducted on four publicly available benchmark datasets widely adopted for biomedical image classification. They comprise mammographic images, pediatric chest X-rays, brain MRI scans, and dermoscopic skin lesion images, thus covering different imaging modalities, classification tasks, and levels of visual complexity. Representative examples from each dataset are shown in Table 2.

### 3.1. Mammography Dataset (CBIS-DDSM)

The mammography dataset is the Curated Breast Imaging Subset of the Digital Database for Screening Mammography (CBIS-DDSM) [32], publicly available through The Cancer Imaging Archive at https://www.cancerimagingarchive.net/collection/cbis-ddsm/ (accessed on 20 August 2026). A JPEG version is also available on Kaggle at https://www.kaggle.com/datasets/awsaf49/cbis-ddsm-breast-cancer-image-dataset (accessed on 20 August 2026). The reprocessed version adopted in this study contains 3032 mammographic images, including 1685 benign and 1347 malignant cases, organized for binary benign/malignant classification, and is publicly available at https://kaggle.com/datasets/9cef8368a7037c7d63324c73fa6384c0e6f9d02e10c2b07a0c37b640dbf17c45 (accessed on 20 August 2026).

### 3.2. Chest X-Ray Dataset

The Chest X-Ray dataset [34,41] is publicly available through Mendeley Data at https://data.mendeley.com/datasets/rscbjbr9sj/2 (accessed on 20 August 2026). Alternative versions are also available on Kaggle at https://www.kaggle.com/datasets/paultimothymooney/chest-xray-pneumonia (accessed on 20 August 2026) and https://www.kaggle.com/datasets/tolgadincer/labeled-chest-xray-images (accessed on 20 August 2026). The version considered in this study contains 5856 pediatric chest radiographs belonging to three classes: Normal (1583), Bacterial Pneumonia (2780), and Viral Pneumonia (1493). The reprocessed datasets used in our experiments are publicly available on Kaggle at https://kaggle.com/datasets/ce5259e7e743223ff2888759297abd27d388cd59af68e7a55ffb25ce8352ab19 (accessed on 20 August 2026, V2 version).

### 3.3. Brain Tumor Dataset

The Brain Tumor Dataset [33] is publicly available on Figshare at https://figshare.com/articles/dataset/brain_tumor_dataset/1512427 (accessed on 20 August 2026), with an alternative version available on Kaggle at https://www.kaggle.com/datasets/nikhilroxtomar/brain-tumor-segmentation (accessed on 20 August 2026). It contains 3064 contrast-enhanced T1-weighted MRI images belonging to three tumor classes: Meningioma (708), Glioma (1426), and Pituitary Tumor (930). The reprocessed version adopted in this study is publicly available at https://kaggle.com/datasets/4e5596da6b1ebfa9044ac3ddf2a424d12968f6ee193ec7d3b02aa31b7247d5df (accessed on 20 August 2026).

### 3.4. ISIC Challenge 2017

The ISIC Challenge 2017 dataset [35] is publicly available through the ISIC Archive at https://challenge.isic-archive.com/data/#2017 (accessed on 20 August 2026). It contains dermoscopic images classified into Melanoma (521), Seborrheic Keratosis (386), and Nevus (1843). The reprocessed version considered in this study comprises 2,750 labeled images and is publicly available on Kaggle at https://kaggle.com/datasets/4e83433e33e4b0eb6fa18f9ad6956a3340d317ac4156d9538ed95704a0db2b62 (accessed on 20 August 2026).

Note: Some datasets may contain multiple images from the same patient, not easily identifiable. Therefore, the train/test split was performed at the image level, potentially inflating accuracy through patient-level leakage. This limitation should be considered when interpreting the reported accuracy values.

## 4. Experimental Design and Sample-Size Reduction Strategy

This section describes the experimental framework adopted to investigate the effect of training-set size on CNN performance across different architectures and biomedical imaging datasets.

### 4.1. Fixed Hyperparameters

To isolate the effects of dataset size and network architecture, all training hyperparameters were held constant throughout the experiments. The adopted configuration is summarized in Table 3. No data augmentation was applied, allowing the direct assessment of the impact of reduced training-set availability without additional variability introduced by artificial transformations. Across repetitions, the training subset was independently resampled, while weight initialization and mini-batch ordering were subject to stochastic variation.

### 4.2. CNN Architectures Considered

The CNN architectures considered in this study are obtained by varying network depth and width, defined respectively as the number of convolutional layers and the number of filters per layer. The explored architectural space includes all combinations of 1–3 convolutional layers with 16, 32, and 64 filters per layer, resulting in 39 distinct configurations evaluated for each dataset.

The complete architectural grid was evaluated under every sample-size condition, enabling a systematic analysis of the interaction between model capacity and training-set availability. The considered configurations span a range of representational capacities, from shallow and narrow networks to deeper and wider architectures, while maintaining the same training procedure across all experiments.

### 4.3. Systematic Variation of Training-Set Size

For each dataset, training subsets of different sizes were independently sampled from the original training set while preserving the original class proportions. Seven training-set fractions were considered:

(3) p∈{5%,10%,20%,40%,60%,80%,100%}.

The test set was kept fixed across all experiments, corresponding to the same stratified 20% subset of the complete dataset, ensuring that models trained with different amounts of data were evaluated on identical unseen samples. The validation set was reduced proportionally with the training fraction.

For each dataset, the 39 CNN architectures were evaluated at the seven training-set fractions, with 5 independent training runs performed for each architecture–sample-size combination. At each fraction, the training subset was independently resampled at each repetition, together with the random seed controlling weight initialization and mini-batch ordering. This experimental design resulted in:

$$\underset{\mathrm{CNN}\;\mathrm{architectural}\;\mathrm{configurations}}{\underset{︸}{39}}\times \underset{\mathrm{training}-\mathrm{set}\;\mathrm{fractions}}{\underset{︸}{\;7\;}}\times \underset{\mathrm{independent}\;\mathrm{runs}}{\underset{︸}{\;5\;}}=1365$$

training runs per dataset, for a total of 5460 experiments across the four datasets.

For each run, classification accuracy was recorded on training, validation, and test sets after the epoch selected by early stopping.

## 5. Sample-Size Sensitivity and Overfitting Analysis

This section describes the analytical framework used to characterize how training-set size affects CNN performance across datasets and architectures. The analysis focuses on three complementary aspects: whether the training–test generalization gap eventually stabilizes as the training-set fraction increases; whether the relative ranking of architectures by test performance, obtained from a reduced training fraction, is a reliable proxy for the ranking obtained on the full dataset; and the monotonicity of test performance with increasing training-set size. Numerical results and figures are presented throughout this section on a per-dataset basis, and synthesized across datasets in Section 6.

### 5.1. Generalization Gap and Overfitting Onset

**Figure 2:**
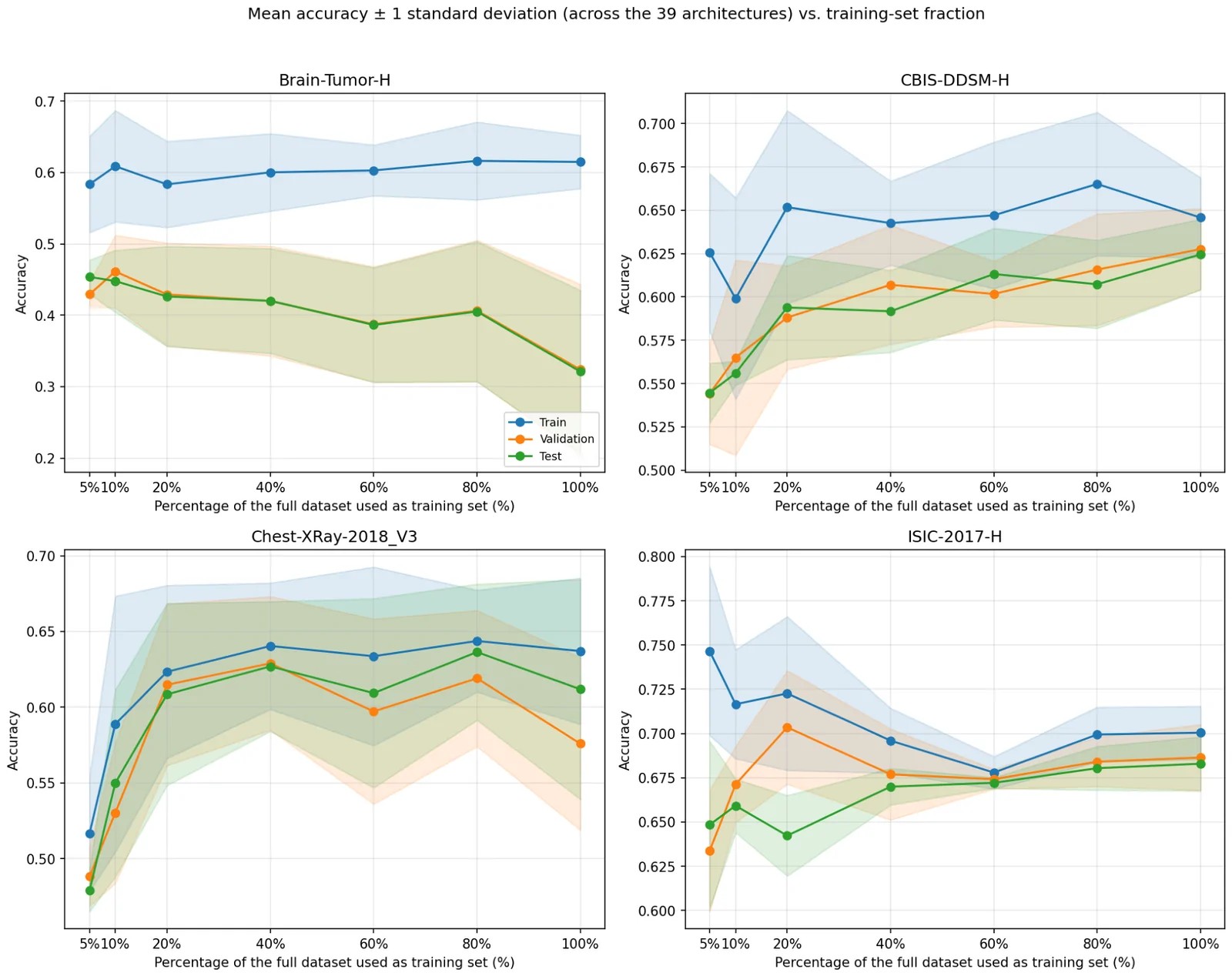
Mean accuracy (±1 standard deviation across architectures and repetitions) for training, validation, and test sets as a function of the training-set fraction *p*.

**Figure 3:**
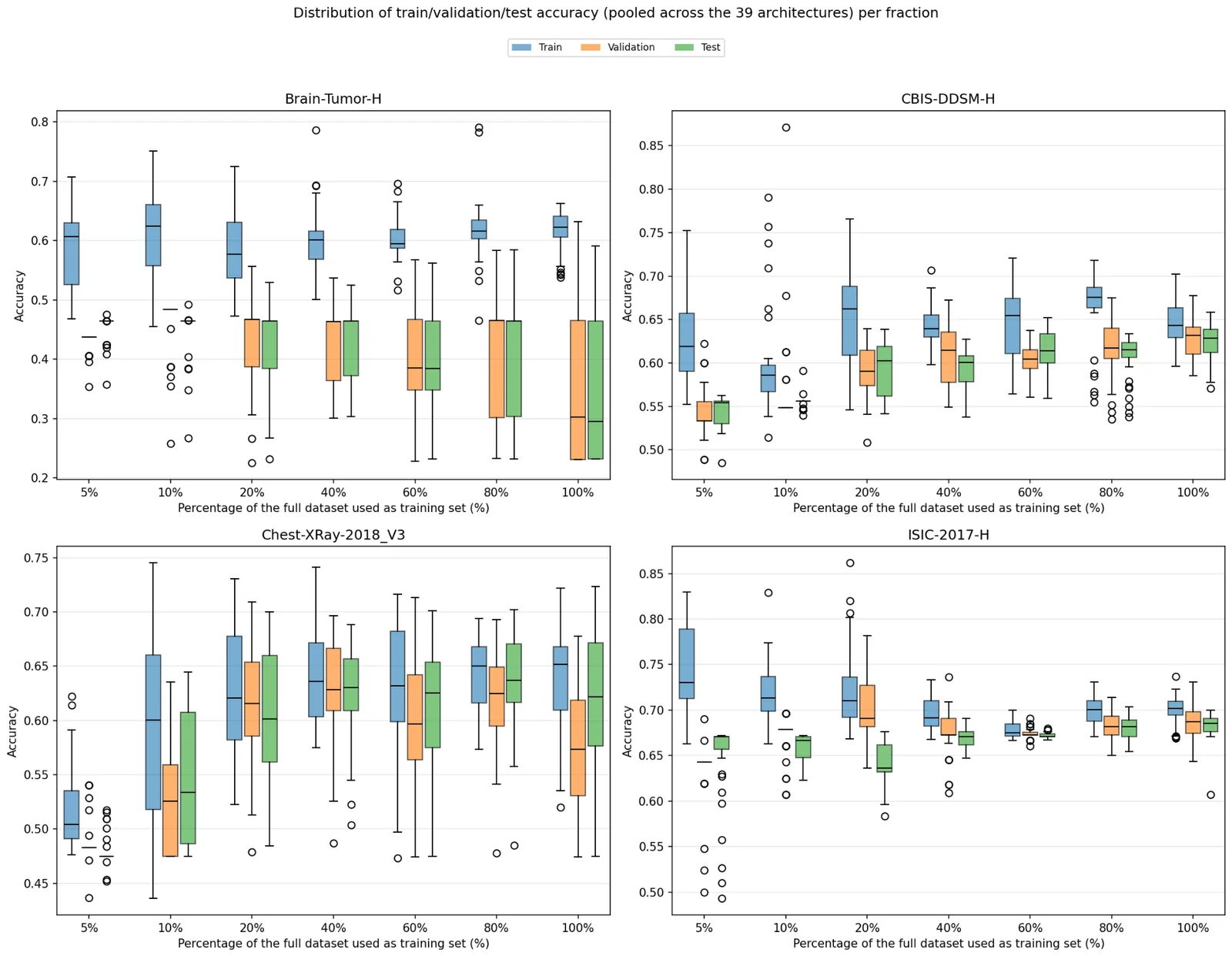
Distribution of training, validation, and test accuracy across architectures and repetitions at each training-set fraction *p*.

Potential overfitting can be assessed by examining whether model performance on the training set evolves differently from performance on the validation and test sets as the training-set fraction *p* increases. A widening discrepancy between training performance and performance on unseen data may indicate increasing overfitting, whereas similar trends across the three sets suggest more consistent generalization. To characterize these patterns, two complementary visualizations are reported below. Figure 2 presents the mean accuracy and standard deviation across the 39 architectures and repetitions for the training, validation, and test sets at each training-set fraction. Figure 3 complements this analysis by reporting the corresponding pooled distributions through boxplots, providing a more detailed view of the variability and dispersion of model performance across architectures and repetitions.

**Figure 4:**
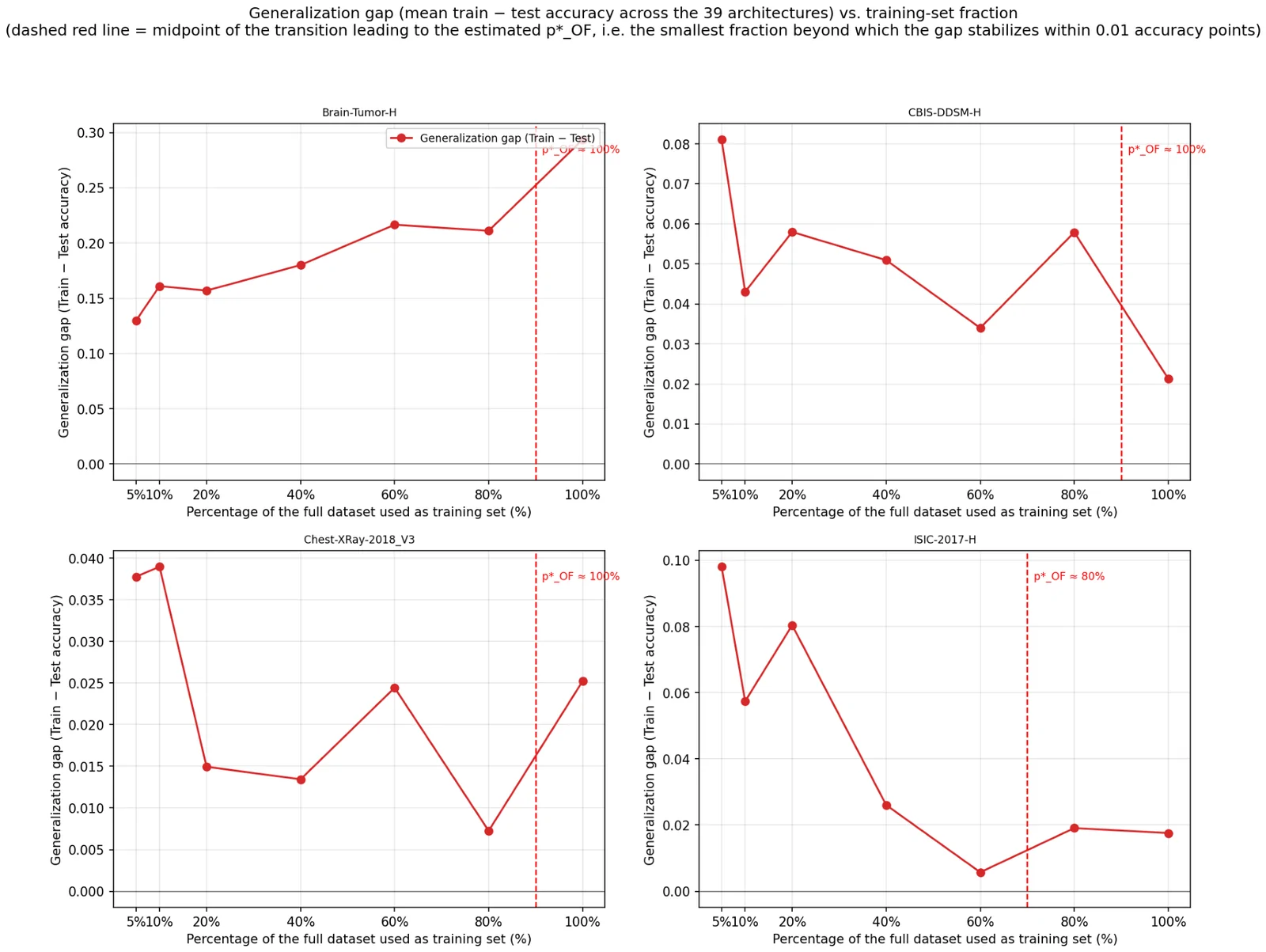
Generalization gap $\Delta_{\mathrm{gen}}$ as a function of the training-set fraction *p* for each dataset. The vertical red line indicates the estimated overfitting-onset threshold $p_{\mathrm{OF}}^{*}$ according to the stabilization criterion with τ=0.01.

To quantify the divergence between training and test performance, we compute the generalization gap for each dataset, architecture, and fraction *p* as:

(4) $$\Delta_{\mathrm{gen}}\left(p\right)={\mathrm{Acc}}_{\mathrm{train}}\left(p\right)-{\mathrm{Acc}}_{\mathrm{test}}\left(p\right),$$

averaged across the 5 independent runs for each architecture, then across the 39 architectures to obtain a dataset-level curve. We define the overfitting-onset threshold $p_{\mathrm{OF}}^{*}$ as the smallest training-set fraction beyond which the generalization gap $\Delta_{\mathrm{gen}}\left(p\right)$ stabilizes. To determine this point, we first compute the absolute change in $\Delta_{\mathrm{gen}}$ between each pair of consecutive training fractions (e.g., between 5% and 10%, between 10% and 20%, and so on up to 80% and 100%). We then identify the smallest fraction such that all subsequent consecutive changes remain below a tolerance of τ=0.01 (one percentage point). This criterion identifies the first fraction beyond which the gap can be considered approximately stable, indicating that further training data no longer substantially alters the generalization behavior. Figure 4 plots $\Delta_{\mathrm{gen}}\left(p\right)$ for all four datasets, with the estimated $p_{\mathrm{OF}}^{*}$ marked by a vertical red line.

### 5.2. Architecture Ranking Reliability

**Figure 5:**
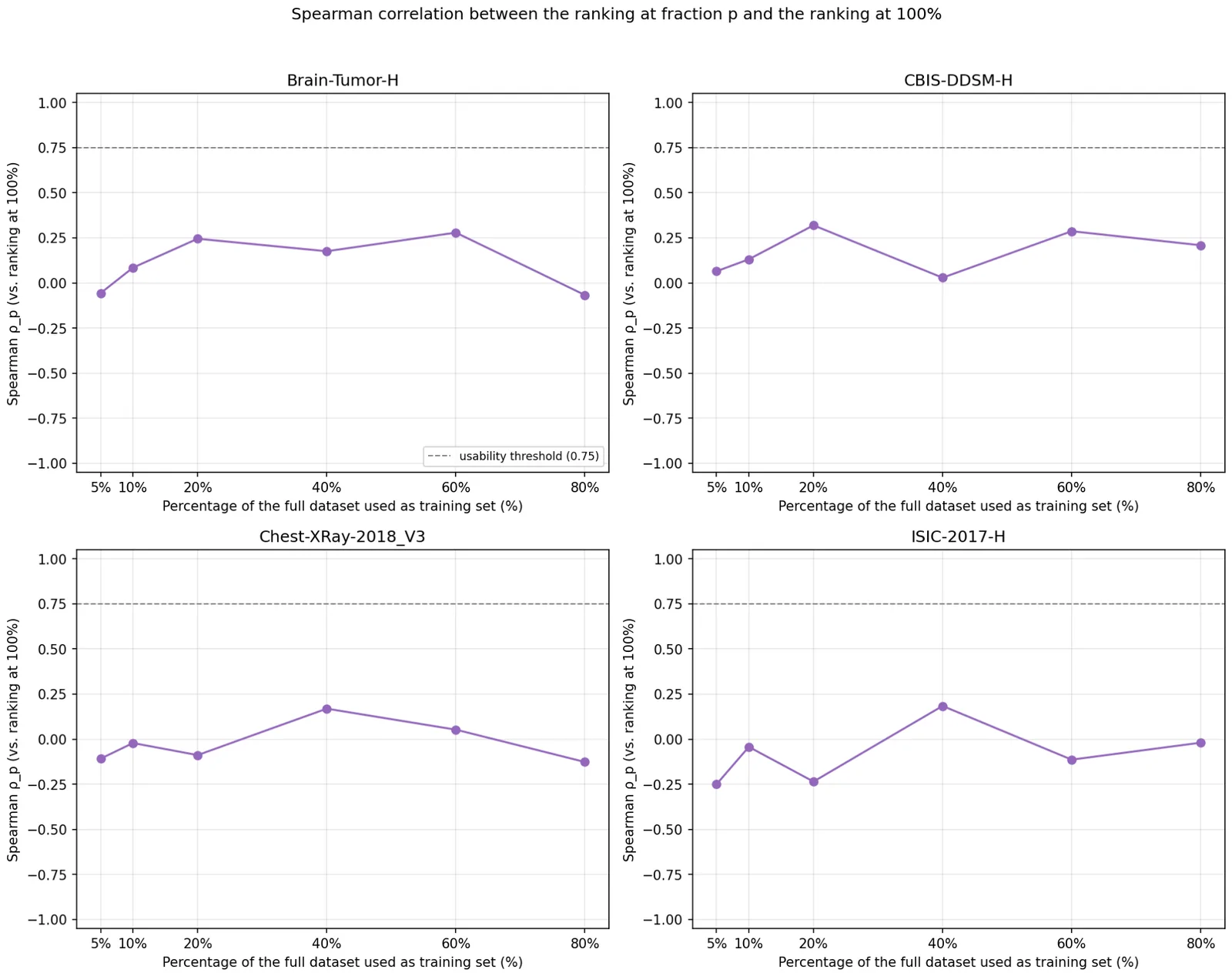
Spearman correlation between the architecture ranking at fraction *p* and the ranking obtained using the complete training set.

Beyond overall performance degradation, an important practical question is whether architectures selected using reduced datasets achieve a ranking consistent with the ranking obtained using the complete training set. The ranking of the 39 architectures at each fraction *p* is therefore compared with the reference ranking obtained at p=100%. For each dataset and fraction, Spearman rank correlation is computed:

(5) $$\rho_{p}=\mathrm{Spearman}\left(R_{p},R_{100\%}\right),$$

where $R_{p}$ denotes the architecture ranking at fraction *p*. The ranking-stabilization fraction $p_{\mathrm{RANK}}^{*}$ is defined as the smallest fraction for which $\rho_{p}$ exceeds a predefined practical usability threshold of $\rho_{p}\geq 0.75$. In simple terms, Spearman rank correlation measures how similarly the 39 architectures are ordered at a given training-set fraction *p* compared with the reference ordering obtained using the complete training set. A value close to 1 indicates that the two rankings are highly consistent, whereas values close to 0 indicate little agreement and negative values indicate that the two rankings tend to be ordered in opposite directions. Therefore, as *p* increases, a high $\rho_{p}$ would indicate that a reduced training set is already sufficient to identify architectures in approximately the same order as the full training set. The threshold $\rho_{p}\geq 0.75$ is used to identify the point at which this agreement becomes sufficiently strong for practical architecture screening. Figure 5 reports $\rho_{p}$ as a function of *p* for each dataset, together with the usability threshold.

**Figure 6:**
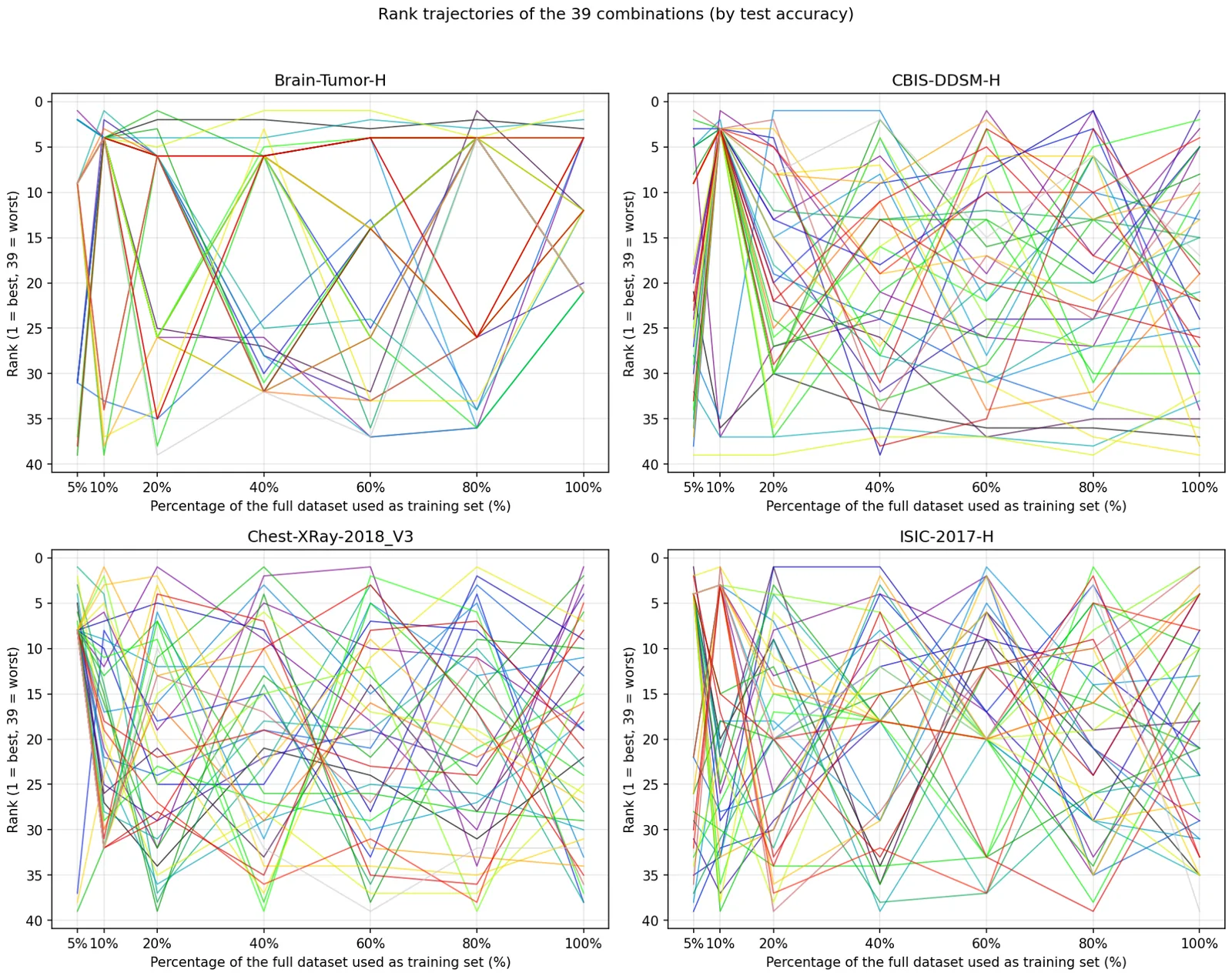
Ranking of the 39 architectures as a function of the training-set fraction *p*.

Figure 6 complements this view by showing the rank trajectories of all 39 architectures as a function of *p*: a stable ranking would appear as approximately parallel trajectories, whereas frequent rank crossings indicate instability.

### 5.3. Test Performance Monotonicity

The previous analyses assume that test performance generally improves as more training data becomes available. This assumption is explicitly evaluated here by checking, for each architecture, whether its test accuracy at p=100% is at least as high as the peak test accuracy reached at any smaller fraction. For architecture *a*, the drop is defined as

(6) $$D_{a}=\max_{p\in \{5\%,\ldots ,100\%\}}{\mathrm{Acc}}_{\mathrm{test}}\left(a,p\right)\;-\;{\mathrm{Acc}}_{\mathrm{test}}\left(a,100\%\right),$$

i.e., the difference between the best test accuracy observed anywhere along the curve and the accuracy obtained using the full training set. A monotonicity violation is flagged whenever $D_{a}$ exceeds a fixed threshold of 0.02 accuracy points (2 percentage points), meaning that using the entire training set produced a test accuracy that is meaningfully worse than what the same architecture had already achieved with less data.

**Figure 7:**
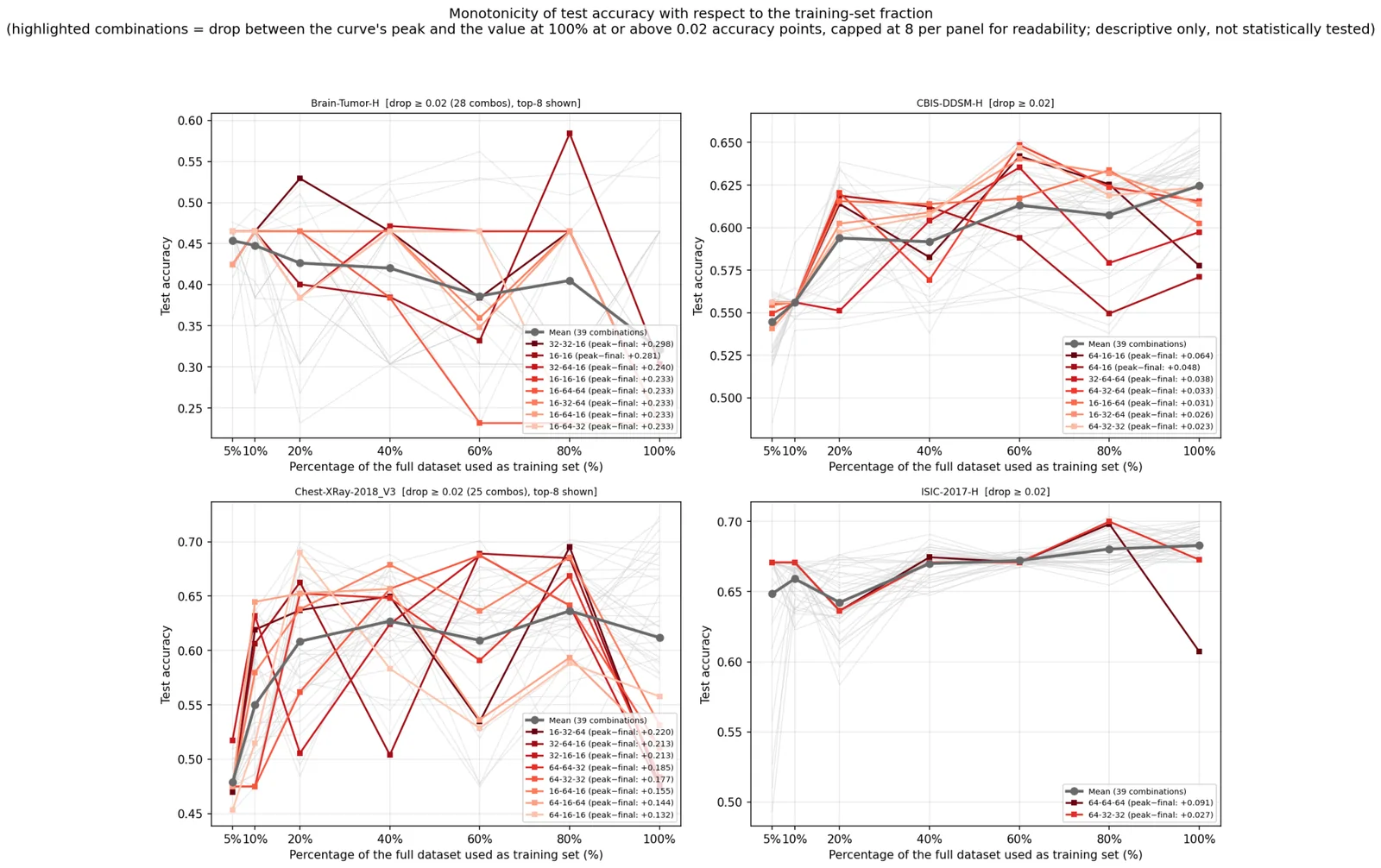
Test accuracy as a function of the training-set fraction *p* for all 39 architectures (thin gray lines) and their mean (dark gray). Architectures whose peak test accuracy exceeds their accuracy at p=100% by at least 0.02 are highlighted in red, with darker shades indicating larger drops; at most the eight largest drops are shown per panel.

Figure 7 shows the test-accuracy trajectory of every architecture (light gray) together with the mean curve across all 39 architectures (dark gray), for each dataset. Architectures whose drop $D_{a}$ exceeds the threshold are highlighted in red, with color intensity proportional to the size of the drop (darker red = larger drop); when more than eight architectures exceed the threshold, only the eight largest drops are highlighted for readability, and the panel title reports the total count.

## 6. Results

This section synthesizes the findings from the overfitting-onset, ranking-stabilization, and monotonicity analyses presented in Section 5, and discusses their practical implications for CNN architecture screening under limited training data. Table 4 summarizes the estimated $p_{\mathrm{OF}}^{*}$ and $p_{\mathrm{RANK}}^{*}$ for each dataset, together with the monotonicity-violation rate discussed in point (iii) below.

*(i)*  Overfitting-onset threshold $p_{\mathrm{OF}}^{*}$

Visual inspection of Figure 2 shows that the expected learning-curve pattern—a flat, high training curve with validation/test curves rising toward it—holds for only ISIC-2017-H, where the three curves become visually indistinguishable from around p=60%, earlier than the tolerance-based estimate of $p_{\mathrm{OF}}^{*}=80\%$ reported in Table 4 (see below for how this value is obtained). Brain-Tumor-H instead shows a flat training curve with validation and test decreasing as *p* grows, consistent with its widening gap; no stabilization point is visible. CBIS-DDSM-H and Chest-X-Ray-2018 show all three curves rising together, keeping an approximately constant separation from p=10% and p=5% respectively; CBIS-DDSM-H at p=5% instead resembles the ISIC-2017-H pattern. Figure 3 shows markedly wider spread and more outliers in test/validation accuracy below p=20% across all four datasets, even where the mean curves in Figure 2 look well-behaved.

Quantitatively, $p_{\mathrm{OF}}^{*}$ is estimated as the smallest fraction beyond which the generalization gap varies by less than the tolerance τ across all subsequent consecutive fractions (Section 5.1). Only ISIC-2017-H shows a genuine stabilization within the sampled range: the generalization gap decreases from 0.098 at p=5% to 0.018 at p=100% and varies by less than τ between p=80% and p=100%, yielding $p_{\mathrm{OF}}^{*}=80\%$. For the remaining three datasets, the gap does not stabilize within p∈[5%,100%]; for Brain-Tumor-H in particular it widens almost monotonically, from 0.130 at p=5% to 0.294 at p=100%—the opposite of the expected trend, and discussed further as a monotonicity violation in point (iii). These qualitative onsets do not match the quantitative $p_{\mathrm{OF}}^{*}$ estimates, which default to p=100% for CBIS-DDSM-H and Chest-X-Ray-2018: a parallel, still-rising band can look qualitatively stable while failing the absolute-tolerance test on the mean gap.

*(ii)*  Ranking-stabilization threshold $p_{\mathrm{RANK}}^{*}$

Figure 5 shows that $\rho_{p}$ does not exhibit a clear increasing trend toward the usability threshold of 0.75 for any of the four datasets: correlations remain low, and are frequently close to zero or negative, across the entire range from p=5% to p=80% (e.g., $\rho_{p}=-0.251$ for ISIC-2017-H at p=5%, $\rho_{p}=-0.127$ for Chest-X-Ray-2018 at p=80%). Figure 6 confirms this instability at the level of individual architectures: rank trajectories are far from parallel and show frequent, large crossings across the entire sampled range, rather than progressively stabilizing as *p* increases. As a result, $p_{\mathrm{RANK}}^{*}$ could not be estimated within the sampled range for *any* dataset: the ranking obtained from a reduced training fraction is not, at any tested fraction, a reliable proxy for the ranking obtained on the full dataset.

*(iii)*  Monotonicity of test performance

Visual inspection of Figure 7 shows that several flagged architectures hold their peak accuracy through p=80% and only collapse in the final 80%→100% step, most visibly in Chest-X-Ray-2018 but also for both flagged architectures in ISIC-2017-H and a subset of those in Brain-Tumor-H—suggesting the last 20% of training data is disproportionately responsible for the observed violations, rather than a gradual decline across *p*.

Quantitatively, monotonicity violations are widespread but highly uneven across datasets. ISIC-2017-H shows the fewest, with only 2 of 39 architectures (5%) exceeding the threshold (largest drop $D_{a}=0.091$, for 64-64-64), consistent with its near-stabilized generalization gap (point (i)); CBIS-DDSM-H shows an intermediate rate, 7/39 (18%), with drops up to $D_{a}=0.064$ (64-16-16). In contrast, Brain-Tumor-H and Chest-X-Ray-2018 show violations for the majority of architectures—28/39 (72%) and 25/39 (64%), respectively—with drops as large as $D_{a}\approx 0.30$ for Brain-Tumor-H (e.g., 32-32-16, $D_{a}=0.298$; 19 architectures sharing an identical drop of $D_{a}=0.233$, all peaking at p=60%), consistent with the monotonic widening of its generalization gap already reported in point (i) and indicating that, for most architectures, training on the full dataset is actively detrimental rather than merely non-beneficial. Chest-X-Ray-2018 shows comparably widespread but more heterogeneous drops, from $D_{a}=0.220$ (16-32-64) down to $D_{a}=0.021$ (16-32-16).

## 7. Discussion

Taken together, the per-dataset evidence discussed in points (i)–(iii) suggests a rough, dataset-dependent four-band heuristic for interpreting how much confidence can be placed in results obtained at reduced training fractions. Below p=20%, results should be treated with considerable caution, as Figure 3 shows markedly wide variability and frequent outliers in test and validation accuracy across all four datasets. Between p=20% and p=60%, variability generally decreases, but it remains unclear whether the test and validation curves are tracking the training curve or whether overfitting persists; CBIS-DDSM-H and Chest-X-Ray-2018 already stabilize within this interval, whereas Brain-Tumor-H shows no comparable stabilization at any fraction. Between p=60% and p=80%, the three curves tend to move together most consistently, and this range includes the only stabilization identified quantitatively in this study, for ISIC-2017-H ($p_{\mathrm{OF}}^{*}=80\%$). Between p=80% and p=100%, using the full training set can be counterproductive for some architectures rather than simply unnecessary, with several flagged architectures holding their peak accuracy through p=80% before declining in the final step (point (iii)).

The behavior observed below p=20% is broadly consistent with what would be expected when the available training sample provides only a limited representation of the underlying data distribution. With fewer observations, the particular composition of the training subset can have a comparatively large influence on the learned model, resulting in greater sensitivity to sampling and optimization stochasticity. This interpretation is consistent with the general behavior of learning curves, for which performance estimates are typically less stable in low-data regimes [20,21]. In the present experiments, this effect is particularly relevant because the training subset was independently resampled at each repetition. Thus, the pronounced dispersion observed at very small fractions is not merely a consequence of differences in parameter initialization, but also reflects the sensitivity of the training process to which observations are available. The fact that this pattern is visible across all four datasets suggests that instability at very small fractions is a general feature of the experimental setting, although its magnitude may depend on dataset-specific characteristics.

The reduction in variability observed between p=20% and p=60% is likewise compatible with the expectation that increasing the amount of training data progressively reduces sensitivity to the particular sampled subset. However, the different behavior of the four datasets indicates that there is no universal point at which increasing the sample size produces stable generalization behavior. The amount of data required to reach such a regime is likely to depend not only on dataset size, but also on the complexity and heterogeneity of the classification problem, the degree of class overlap, and the interaction between model capacity and the information contained in the available observations. Previous work on learning curves and sample-size effects similarly emphasizes that the relationship between additional training data and generalization depends on the learning setting rather than following a single universal trajectory [20,21]. The absence of stabilization for Brain-Tumor-H therefore suggests that a numerically substantial training fraction should not automatically be interpreted as sufficient evidence of stable generalization.

The comparatively regular behavior observed between p=60% and p=80% can be interpreted as a regime in which the training subsets are large enough to reduce much of the sampling variability evident at smaller fractions, while the non-monotonic effects observed in the final increment toward 100% have not yet become prominent. This interpretation is compatible with the general expectation that the marginal effect of additional training observations can diminish as the sample becomes sufficiently informative [20,21]. Importantly, however, stability of the generalization gap should not be confused with stability of architecture selection. Even in the comparatively stable regime, the architecture rankings did not reliably approach the full-data ranking. This distinction is consistent with research showing that the usefulness of reduced-data or low-fidelity evaluations for architecture comparison depends on the extent to which rankings are preserved across evaluation budgets [13,15]. In other words, a training fraction may provide a relatively stable estimate of generalization behavior without providing a reliable basis for deciding which architecture is preferable.

The behavior observed between p=80% and p=100% is more difficult to interpret and should not be regarded simply as evidence that additional data cause overfitting. Rather, the results show that increasing the training fraction does not guarantee a corresponding improvement in test accuracy for every architecture–dataset combination. Such non-monotonic relationships between sample size and generalization have been reported in previous studies, indicating that the benefit of additional data need not be uniformly monotonic under all learning conditions [16,17,18,19]. In the present setting, the concentration of several violations in the final 80%→100% increment suggests that the effect may depend on the interaction between the additional observations, the architecture, and the optimization procedure. However, the present design does not allow these possible mechanisms to be separated, and therefore does not establish a specific causal explanation for the observed degradation. The result is better interpreted as evidence that the assumption of monotonically improving test performance should be empirically checked rather than taken for granted.

Taken together, the four bands should be interpreted as dataset-dependent regimes rather than universal thresholds. Lower fractions are mainly characterized by high variability, intermediate fractions by progressively more stable but still dataset-dependent generalization behavior, and higher fractions by the possibility of diminishing or even negative returns from additional data for some architecture–dataset combinations. Importantly, these aspects of reliability do not necessarily emerge together: reduced variability does not imply a stable generalization gap, a stable gap does not imply a stable architecture ranking, and increasing the training set does not guarantee improved test performance. This helps explain why no single reduced training fraction could be identified as a generally reliable shortcut for CNN architecture selection. Training-set size should therefore be considered not only as a computational constraint but also as an experimental factor affecting the reliability and interpretation of model comparisons, with evaluation across multiple training-set sizes providing a more informative view of an architecture’s effective operating range and of the robustness of conclusions about performance, ranking, and generalization.

## 8. Conclusions

This study systematically evaluated 39 CNN architectures across four biomedical imaging datasets (mammography, pediatric chest X-ray, brain tumor MRI, and skin lesion dermoscopy), each trained from scratch on seven training-set fractions with 5 independent repetitions, to assess three complementary aspects of sample-size sensitivity: the stabilization of the training–test generalization gap, the reliability of architecture rankings obtained from reduced training fractions, and the monotonicity of test performance with respect to training-set size.

The results point to a rough, dataset-dependent four-band pattern of reliability: results obtained below p=20% show markedly high variability across all four datasets; between p=20% and p=60% the overfitting status remains uncertain, with two datasets (CBIS-DDSM-H, Chest-X-Ray-2018) already stabilizing in this range while Brain-Tumor-H never stabilizes; between p=60% and p=80% the generalization gap is comparatively most stable, including the only genuine stabilization observed in this study (ISIC-2017-H, $p_{\mathrm{OF}}^{*}=80\%$); and beyond p=80%, using the full training set is potentially counterproductive, with monotonicity violations affecting the majority of architectures on two of the four datasets (72% for Brain-Tumor-H, 64% for Chest-X-Ray-2018). Critically, this pattern offers no guarantee on architecture ranking: no training-set fraction was found, for any dataset, at which the ranking of the 39 architectures reliably matched the full-dataset ranking, arguing against reduced-fraction screening as a dependable shortcut for CNN architecture selection in biomedical imaging.

## Figures and Tables

**Table 1:** Illustration of the diagonal true-positive elements in a three-class confusion matrix, boxed to highlight the correctly classified samples forming the numerator of accuracy, while the denominator represents all samples.

	Class A	Class B	Class C
Class A	$\begin{array}{c} TP_{A} \end{array}$		
Class B		$\begin{array}{c} TP_{B} \end{array}$	
Class C			$\begin{array}{c} TP_{C} \end{array}$

**Table 2:** Overview of the biomedical datasets considered in this study and representative examples of the corresponding classes.

Dataset	Class		
	Benign	Malignant	
Mammography Dataset (CBIS-DDSM) 3032 images total Benign: 1685 Malignant: 1347	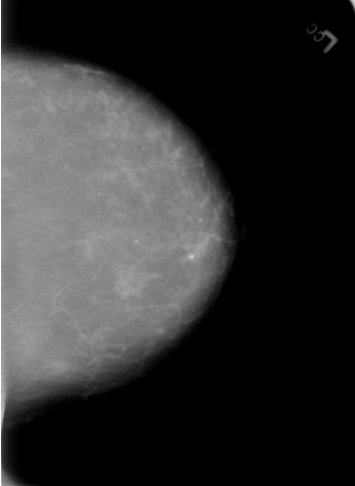	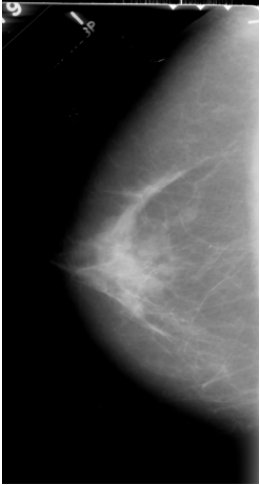	
Chest X-Ray Dataset	Normal	Bacteria	Virus
5856 images total Normal: 1583 Bacteria: 2780 Virus: 1493	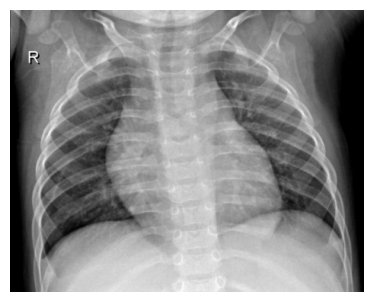	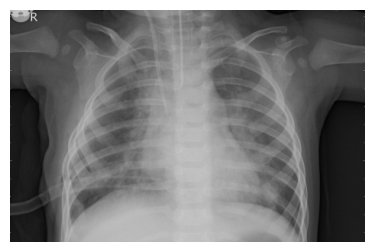	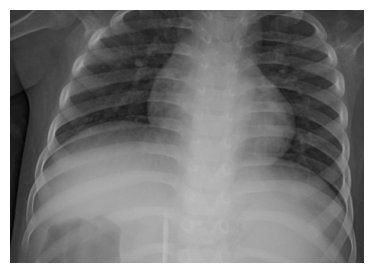
Brain Tumor Dataset	Meningioma	Pituitary Tumor	Glioma
3064 images total Meningioma: 708 Pituitary Tumor: 930 Glioma: 1426	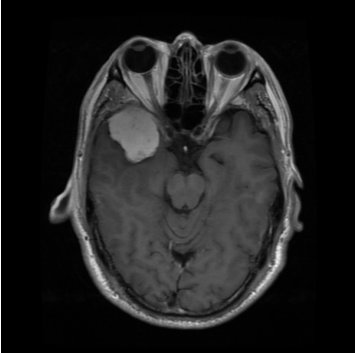	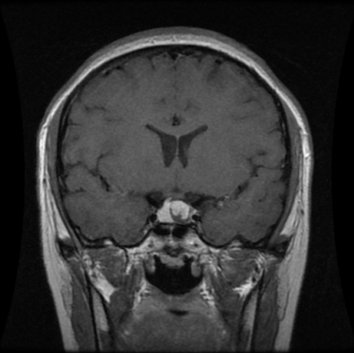	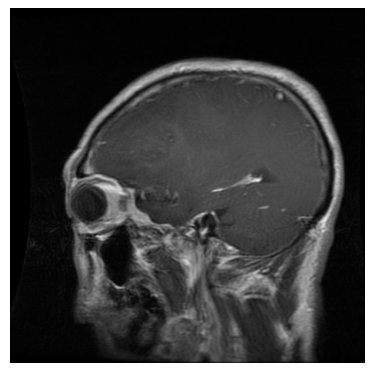
ISIC Challenge 2017	Melanoma	Seborrheic keratosis	Nevus
2750 images total Melanoma: 521 Seborrheic keratosis: 386 Nevus: 1843	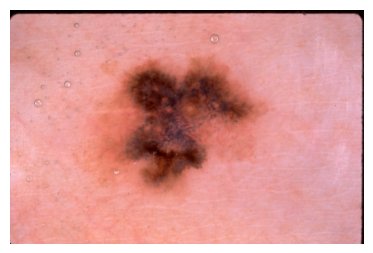	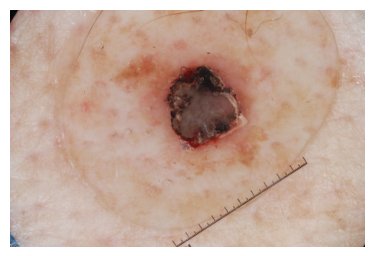	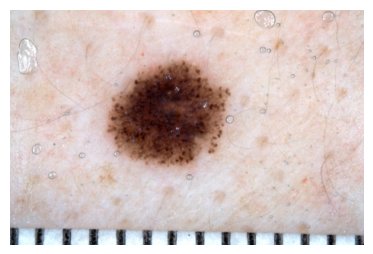

**Table 3:** Hyperparameters adopted for all CNN training experiments.

Parameter	Value
Dataset Split	70% Train, 10% Validation, 20% Test
Data Augmentation	None
Input Normalization	[0, 1] range
Input Size	256×256 pixels
Convolutional Filter Size	3×3
Padding	same, stride 1
Batch Normalization	Applied
Activation Function	ReLU
Pooling	MaxPooling stride 2; GlobalAveragePooling2D()
Fully Connected Layer	256 neurons
Dropout	0.3
Optimizer	Adam
Learning Rate	$1\times 10^{-3}$
Batch Size	16
Loss Function	Sparse Categorical Crossentropy
Epochs	100 with early stopping after 20 epochs

**Table 4:** Summary of the estimated overfitting-onset threshold $p_{\mathrm{OF}}^{*}$, ranking-stabilization threshold $p_{\mathrm{RANK}}^{*}$, and the number of architectures (out of 39) violating monotonicity, per dataset. An asterisk denotes a threshold not genuinely reached within the sampled range (see point (i) below).

Dataset	$p_{\mathrm{OF}}^{*}$	$p_{\mathrm{RANK}}^{*}$	Monotonicity Violations
Brain-Tumor-H	100% *	n.d.	28/39 (72%)
CBIS-DDSM-H	100% *	n.d.	7/39 (18%)
Chest-X-Ray-2018	100% *	n.d.	25/39 (64%)
ISIC-2017-H	80%	n.d.	2/39 (5%)

## Data Availability

All data generated or analyzed during this study are available at https://github.com/hyacintus/CNN-Architectural_Design-Biomedical-Datasets.git (accessed on 20 August 2026).
